# Teff grass for continuous stocking in the Southern High Plains by growing beef steers receiving protein supplements^1^

**DOI:** 10.1093/tas/txab136

**Published:** 2021-08-18

**Authors:** Joel D Sugg, Jhones O Sarturi, Charles P West, Michael A Ballou, Darren D Henry

**Affiliations:** 1 Department of Animal and Food Sciences, Texas Tech University, Lubbock, TX, USA; 2 Department of Plant and Soil Sciences, Texas Tech University, Lubbock, TX, USA; 3 Department of Veterinary Sciences, Texas Tech University, Lubbock, TX, USA

**Keywords:** annual forage, beef steers, digestibility, teff grass, water

## Abstract

This experiment evaluated forage quality, total nutrient yield, water footprint, and growth performance of beef steers receiving protein supplements while grazing Teff grass [‘Tiffany’*Eragrostis tef* (Zucc.) Trotter] over two consecutive growing seasons. Each year, four 2.66-ha irrigated paddocks (experimental units) were stocked with crossbred beef steers (*n* = 5 per paddock, initial BW = 289 ± 30 for yr 1; and *n* = 6, initial BW = 286 ± 23 for yr 2) in a randomized complete block design and stocked continuously for 63 d. Daily supplements [0.45 kg/d of cottonseed meal (Control) enough to avoid a negative ruminal N balance; and 0.50% mean paddock BW animal-daily (approximately 1.65 kg) of sorghum-dried distillers grains plus solubles, (DDGS)] were randomly assigned to two paddocks each. Supplement did not influence forage neutral detergent fiber (NDF), acid detergent fiber, crude protein, or in vitro true digestibility (*P* ≥ 0.54), except for a tendency (*P* = 0.08) for a numerical increase in NDF content of paddocks with steers that received DDGS supplementation. Paddock nutrient-yields were similar (*P* ≥ 0.43) between supplement treatments. Supplementation with DDGS produced greater (*P* = 0.01) cattle shrunk average daily gain (ADG). Predicted teff dry matter intake (DMI), net energy for maintenance (NE_m_), and growth (NE_g_) (*P* ≤ 0.03) were greater with cattle offered Control treatment. Predicted total DMI was similar (*P* = 0.14) although predicted dietary NE_m_, NE_g_, gain:feed, and total BW gain were greater (*P* ≤ 0.02) with DDGS. Predicted forage intake was greater (*P* ≤ 0.05) for cattle offered Control treatment. Teff nutrients remaining on d 56 were similar (*P* = 0.33) between treatments. Water footprint for total production of forage nutrient components did not differ (*P* ≥ 0.12) by treatments. Nutrient yield and water use efficiency of continuously stocked teff grass was not affected by supplemental regimen. Using DDGS as a supplement may increase BW gain through increased nutrient utilization without hindering teff nutrient production on a continuous stocking system.

## INTRODUCTION

Summer forage production on the Southern High Plains is restricted by a strong deficit in rainfall relative to high evapotranspiration potential (Tolk and Howell, 2003). Overcoming that deficit necessitates irrigation; however, declining groundwater supplies from the underlying High Plains Aquifer limits water availability (Haacker et al., 2016). As a result, regional backgrounding of growing beef calves is hindered by unreliable availability of warm-season, annual grasses during late summer and early fall. Teff grass (*Eragrostis tef* [Zucc.] Trotter) is an annual C_4_ species which has demonstrated potential to produce high-quality forage for grazing and hay production (Norberg et al., 2009; Miller, 2011; Staniar et al., 2010). Additionally, teff is a vigorous species requiring minimal input, and this contributes to its value as an emergency crop (Assefa et al., 2001; Evert et al., 2009).

Potential for teff to support grazing beef cattle is supported by total forage yield estimates as high as 16 t/ha with multiple harvests within a growing season (Miller, 2011), while protein content may vary throughout the grazing season and soil conditions. Supplementation with dried distillers grains plus solubles (DDGS) may enhance growth performance of grazing animals through retained energy (Morris et al., 2005, 2006) and mitigation of metabolizable protein deficiency (MacDonald et al., 2007). Supplementation with DDGS can cause reduction in voluntary forage intake, likely as a response to sustained low rumen pH and reduced fiber digestion (Loy et al., 2007; Sugg et al., 2021). As a result, supply of a low-input, drought-tolerant forage such as teff could potentially be extended without compromising grazing animal output.

The concept of water footprint quantifies the volume of water inherent in the production of a commodity and is therefore useful for assessing the impact of forage and animal management practices on the conversion efficiency of limited water resources (West and Baxter, 2018). One method of calculating water footprint for beef stocker production is to express the volume of rain received plus irrigation applied per kilogram of live-weight gain (Baxter et al., 2017).

This two-year experiment evaluated estimated forage nutrient production, water footprint, total animal weight gain, and teff forage characteristics during continuous stocking by beef steers receiving a protein supplement (DDGS) while also providing enough protein (cotton seed meal) for the Control treatment, over 63-d summer grazing periods on the Southern High Plains.

## MATERIALS AND METHODS

All procedures involving the use of live animals were approved by the Texas Tech University Institutional Animal Care and Use Committee (IACUC # 15049-06).

### Animals and Treatments

For both years of assessment, Angus crossbred steers (*n* = 20, initial BW = 289 ± 30 kg, yr 1; *n* = 24, initial BW = 286 ± 23 kg, yr 2) were sourced from a commercial cooperator near Stratford, TX (410 km), and used in a randomized complete block design. Prior to arriving at the study site, steers were vaccinated for bovine rhinotracheitis virus, parainfluenza 3-respiratory syncytial virus, *Mannheimia haemolytica*, and *Pasteurella multocida* (Vista Once SQ, Intervet, Inc., Omaha, NE); *Clostridium chauvoei-septicum-novyl-sordellii-perfringens* types C & D bacterin-toxoid (Vision 7 with SPUR, Intervet, Inc., Omaha, NE); as well as treated with an internal and external parasiticide (Safe-guard, Intervet, Inc., Millsboro, DE; and Noromectin Pour-on, Norbrook Laboratories Ltd, Newry, Northern Ireland). Approximately 14 d prior to grazing in both years, steers were group fed a perennial hay [WW-B Dahl’ Old World bluestem *Bothriochloa bladhii* (Retz).T. Blake] add libitum as well as had free access to a mineral supplement packet, which preceded the initial body weight measurement and the initiation of grazing phases. In such period, animals also received a single-dose implant containing 100 mg trenbolone acetate and 14 mg estradiol benzoate (Synovex Choice, Zoetis). Following a 12-h withdrawal from feed and water, individual shrunk body weights were collected at 21-d intervals from d 0 to 63 of grazing in order to quantify average daily gain (ADG) calculated as weight gain within each period divided by days. Immediately after obtaining weights on d 0 of both years, steers were blocked by BW and randomly assigned to paddock (experimental unit; 2/treatment each year). Each paddock was stocked with 5 and 6 steers in yr 1 and 2, respectively.

Paddocks were randomly assigned one of two daily supplements provided in self-fed form in feeders measuring approximately 2.4 m × 0.3 m. Supplement treatments consisted of either crude protein (CP) alone (0.45 kg dry matter [DM] animal-daily cottonseed meal; Control) or an energy-containing byproduct (0.50% mean paddock BW/animal-daily; sorghum-DDGS). Cottonseed meal was provided in quantity enough to provide a positive ruminal N balance (NASEM, 2016) in lieu of a negative control to ensure that adequate dietary nitrogen was available for forage digestion. In addition to supplements, all paddocks were supplied a commercial monensin-containing pasture mineral (Hi-Pro #10230, Hi-Pro Feeds Inc., Friona, TX) with a target consumption of 0.11 kg DM animal-daily to supply 200 mg monensin sodium per animal-daily. Mineral was provided in a separate feeder from the supplement. On alternating days, residual mineral was measured to quantify consumption and feeders replenished with fresh mineral. Both supplements were entirely consumed at each feeding.

### Forage Management and Sampling

Four adjacent 2.66-ha paddocks (experimental unit) located 10.3 km east of New Deal, TX consisting of Pullman clay loam soils and equipped with subsurface drip irrigation were seeded in ‘Tiffany’ teff on June 11–12 and April 25–26 of 2015 (yr 1) and 2016 (yr 2), respectively. Preparation of seedbeds included only light disking prior to seeding. Seeds were no-till drilled at a depth of 0.6 cm and at a rate of 3.7 and 5.9 kg/ha pure live seed in yr 1 and 2, respectively. Grazing was initiated 51 d after seeding in yr 1 and 81 d after seeding in yr 2. Neither herbicide nor fertilizer was applied to the test area in yr 1. In yr 2, a broadleaf herbicide (2, 4-dichlorophenoxyacetic acid; 1.49 L active ingredient/ha) was applied by a commercial applicator 14 d prior to seeding and each paddock received 33.6 kg/ha of nitrogen (N) as urea-ammonium nitrate via irrigation approximately 117 d after seeding.

Beginning at d 0 of grazing and at 7-d intervals for the duration of the grazing period, entire canopy samples within 1 m^2^ quadrats (*n* = 6 per paddock) tossed at random were clipped to 3-cm stubble height to determine DM and organic matter (OM) contents. Samples were also analyzed to quantify detergent fiber concentrations at 14-d intervals beginning on d 7. At each collection, samples (*n* = 6 per paddock) of teff canopy that visually simulated the grazed portion were also collected for the same analyses to describe the difference between total available nutritive value and that of the apparent grazed canopy portion. To sample the grazed canopy portion, a visually apparent, ungrazed patch was clipped to the same stubble height as an adjacent grazed patch. At 14-d intervals, the forage within quadrates was used to determine forage availability and were analyzed for CP as well as in vitro true dry matter (IVTDMD) and organic (IVTOMD) matter digestibility.

Soil volumetric water content was recorded beginning on d 0 of grazing and at 7-d intervals throughout the grazing period using a PR2 Soil Moisture Profile Probe (Delta-T Devices, Cambridge, UK) as described by Dhakal et al. (2019). Moisture was recorded at depths of 100, 200, 300, 400, 600, and 1000 mm at three evenly-spaced locations spanning each paddock.

### Utilization and Input Calculations

For each 21-day period, estimates of teff dry matter intake (DMI), energy values, and total feed efficiency were calculated from observed supplement intake and gain performance using the Beef Cattle Nutrient Requirements Model (NASEM, 2016). Model descriptions of management, environment, and animal were updated to for each period iteration. Briefly, supplement intakes were defined and teff DMI was assumed to equal the model total predicted intake minus offered supplement and mineral packet intakes. Teff total digestible nutrients (TDN) values were adjusted such that metabolizable energy (ME) for gain aligned with actual average daily gain (ADG) observed. Calculated dietary net energy (NE) for maintenance (NE_m_) and growth (NE_g_) values were used to determine NE per kg from teff by removing supplement and mineral from the diet calculation and dividing the remaining NE_m_ and NE_g_ by the model-predicted teff DMI.

Total output was described as calculated teff nutrients consumed in addition to nutrient concentrations of teff remaining at the end of the grazing period. Nutrients consumed were estimated as a function of predicted teff intake and mean nutritive values across the grazing periods. Forage was not sampled following steer removal on d 63. As a result, teff sampled at d 56 was used to describe residual mass and nutritive yield. References to animal unit days (AUD) assume that one animal unit is equivalent to 453.5 kg of shrunk BW. Values for AUD are then calculated as:


$$\begin{array}{ll} AUD\;= & \;\left(\frac{mean\;paddock\;shrunk\;BW}{453.5\;kg}\right)\\ & \times steers\;per\;paddock\;\times \;grazing\;days \end{array}$$

where, mean paddock shrunk BW is calculated for each steer as the mean of initial and final shrunk BW.

**Figure 1.**
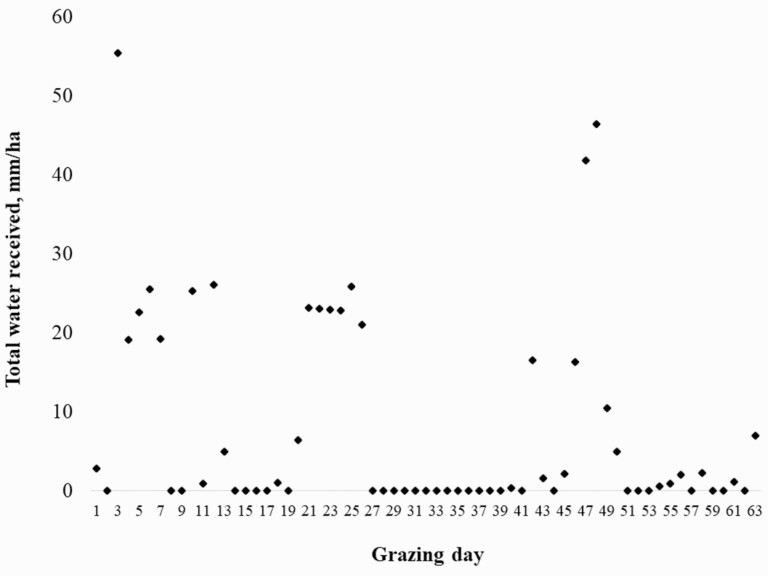
Mean total water depth received (irrigation plus precipitation) by the teff paddocks during the 63-d grazing periods.

Volume of water received per paddock was derived from metered irrigation plus total rainfall during the 63 d of grazing in both years (Figure 1). Rainfall was measured at a single location at the research site and measure presumed to be uniform across all paddocks. Estimates of water use efficiency were determined as total plant output from above relative to m^3^ of water received (irrigation plus rainfall).

### Laboratory Analyses

Samples of entire plant structure as well as non-grazed canopy samples were dried in a forced air oven (55 °C) for 72 h and subsequently ground to pass a 1-mm screen in a Wiley mill (Thomas Scientific, Swedesboro, NJ). Laboratory-corrected DM was determined by further drying samples (0.5 g) in a forced-air oven at 100 °C for 4 h (AOAC, 1990). OM was calculated from ash residue remaining after complete combustion in a muffle furnace at 600 °C for 4 h (AOAC, 1990). CP was measured as 6.25 × percentage of nitrogen as quantified using a Leco CNS Nitrogen Analyzer (Leco CNS-200, St. Joseph, MI). The NDF and ADF concentrations were obtained using filter bag techniques (Ankom Technology, 2006a, 2006b), with the addition of alpha-amylase, sodium sulfite, and subtracting ash from residue. In vitro true digestibility measures were determined using a Daisy^*II*^ Incubator (Ankom Technology, 2005). Rumen content inoculum (combined with buffer [4:1]) used in IVTDMD procedures was composited from 2 ruminally cannulated beef cows (BW = 350 kg ± 11 kg), consuming approximately 8.0 kg DM teff grass hay per day.

### Statistical Analyses

Data were analyzed as a randomized complete block design using the GLIMMIX procedure of SAS 9.4 (SAS Inst., Inc., Cary, NC). Paddock served as experimental unit in all analyses. The model for forage CP, NDF, ADF, and IVTDMD included the fixed effects of supplement type, day, canopy component (whole plant versus the apparent grazed portion), and their interactions. Supplement type, day, and their interactions were included as fixed effects for forage mass. Fixed effects in analysis of soil moisture included supplement, day, depth, and their interactions. Supplement served as the model fixed effect for steer ADG. Fixed effect denominator degrees of freedom were corrected by applying a Kenward-Rogers adjustment. Year was included as a random effect for all variables. In analysis of soil moisture, year (location) was included as the random effect. For repeated measure variables, year × paddock was included as subject statement, and the covariance structure best fit was selected based on the lowest Akaike’s Information Criterion. Interactions were dropped from the model when not significant (alpha > 0.05). Differences were compared using LSMEANS and Tukey’s option for means adjustment was applied when multiple comparisons (i.e., grazing day and soil depth) were made. Results were considered significant at *P ≤* 0.05 and tendencies discussed at 0.05 *< P ≤* 0.10.

## RESULTS AND DISCUSSION

### Forage Quality and Yield

No interactions related to supplement type, canopy component, or grazing day were observed (*P* ≥ 0.36) for teff CP concentration (Table 1). Concentration of CP in the teff was unaffected by supplement type though concentration was greater (*P* < 0.01) in the grazed canopy tissue compared to the entire canopy. CP on d 28 tended (*P* = 0.07) to be lesser than on d 56.

**Table 1.** Teff nutritional composition and in vitro digestibility during the 63-d grazing periods as affected by beef steers supplement type, canopy component, and grazing day

Item		CP^1^, %	NDF^1^, %	ADF^1^, %	IVTDMD^2^, %
Supplement type^3^					
	Control	9.52	67.27	29.83	65.7
	DDGS	9.05	68.32	29.92	65.1
	SEM	0.75	0.57	0.43	1.20
Canopy component					
	Entire canopy	8.49	68.90	30.45	66.0
	Grazed canopy	10.08	66.60	29.30	64.7
	SEM	0.33	0.33	0.24	0.90
Grazing day					
	0	8.88	–	–	66.0
	7	–	69.24^a^	28.73^a^	–
	14	8.65	–	–	65.2
	21	–	67.80^ab^	29.46^ab^	–
	28	7.87	–	–	63.3
	35	–	68.05^ab^	30.46^b^	–
	42	9.9	–	–	66.2
	49	–	66.08^b^	30.87^b^	–
	56	11.08	–	–	66.3
	SEM	1.19	0.81	0.60	2.0
*P*-values					
	Supplement type	0.54	0.08	0.84	0.63
	Canopy component	<0.01	<0.01	<0.01	0.18
	Grazing day	0.09	<0.01	<0.01	0.50
	Supplement type × canopy component	0.72	0.71	0.80	0.94
	Supplement type × grazing day	0.99	0.93	0.81	0.93
	Canopy component × grazing day	0.36	0.07	0.02	0.54
	Supplement type × canopy component × grazing day	0.97	0.91	0.45	0.85

^1^Fiber concentrations quantified at 7-d intervals; and CP and IVTDMD measures quantified at 14-d intervals.

^2^In vitro true dry matter digestibility.

^3^Control: cottonseed meal, 0.45 kg DM/animal-daily; DDGS: dried distillers grains plus solubles, 0.50% mean paddock BW/animal-daily.

^a,b^Within column, means without a common superscript differ at *P* ≤ 0.05.

Regarding N fertilization of teff, Hunter et al. (2010) applied 56 kg N/ha to teff grown for hay and observed a 51 and 62% increase in CP in second and third harvests, respectively, relative to the initial harvest. According to Hunter et al. (2010), N applications above 56 kg/ha per harvest did not result in increased yield and application of N fertilizer may not be necessary to generate additional yield when teff is grazed due to manure deposition. Paddocks in the current study had not been grazed for at least 2 years prior. The rate of nitrogen applied during yr 2 of the current experiment was well within the range of applications reported in the literature. Habtegebrial et al. (2007) cautioned that applying higher rates (90+ kg/ha) of N to teff could increase the risks of N leaching and crop lodging; however, lodging would not be a concern under continuous stocking.

Vinyard et al. (2018) produced ‘Moxie’ teff hay in Idaho without the use of nitrogen fertilizer despite a pre-experiment soil test which recommended 56 kg N/ha. Those authors observed the anticipated decline in CP with maturity at concentrations of 18.7%, 14.7%, and 11.9% at the boot, early-heading, and late-heading stages, respectively. While these values provide reference at each stage of growth, they are not ideal for comparison to those in the current experiment due to the regenerative growth associated with constant grazing.

With the exception of grazing component × day (NDF, *P* = 0.02; ADF, *P* = 0.05), no two-way or three-way interactions of supplement type, grazing component, or grazing day were observed (NDF, *P* ≥ 0.71; ADF, *P* ≥ 0.45) for fiber concentration of teff. A tendency was observed (*P* = 0.08) for reduced NDF in paddocks assigned Control treatment, though ADF (*P* = 0.87) was similar. Staniar et al. (2010) reported that in teff hay harvested at multiple stages, NDF remained constant through the early-heading stage whereas ADF increased significantly between the boot and early-heading stages. In a similar study, Vinyard et al. (2018) reported that both NDF and ADF concentrations in teff hay remained constant from the boot stage to the late-heading stage with means of 62.3% and 28.1%, respectively. However, as with the CP discussion, comparison of results from the current experiment and those in the literature are not advised due to grazing versus progressive maturity systems.

Calculated as NDF minus ADF, the concentration of hemicellulose throughout the grazing period in the current study ranged from 28.59% to 40.50% and from 28.43% to 40.54% in the entire canopy structure and canopy, respectively. Hemicellulose concentrations at d 49 and 56 were intermediary to overall observed ranges. As a result, increases in detergent fiber concentrations at the end of the period are presumed to reflect increased cell wall mass and the divergence between entire canopy and canopy structures attributed to greater proportions in the stalk.

No interactions of supplement type, grazing component, or grazing day were observed for IVTDMD (*P* ≥ 0.54). Consistent measures of IVTDMD across the growing season align with the observations of Vinyard et al. (2018) who observed no increase in cellulose, lignin, or ADF in teff hay harvested at multiple stages of maturity. However, Staniar et al. (2010) evaluated teff hay at the same stages of growth and reported increased NDF in the late-heading stage and greater ADF in the early-heading and late-heading stages. Possible explanations for these discrepancies may include differences in cultivar, climate, or a slightly later harvest date in the latter study.

Available forage mass was estimated at 14-d intervals to characterize seasonal variation. Forage DM or OM mass did not produce a grazing day × supplement type interaction (both, *P* = 0.83). Similarly, no effects of either grazing day (both DM, *P* = 0.38; OM, *P* = 0.39) or supplement type (DM, *P* = 0.43; OM, *P* = 0.46) were detected (Table 2). When BW means within supplement treatment in the current experiment were pooled and interpolated within 21-d intervals to align with days when forage availability was quantified, forage DM allowance at the respective 14-d intervals was calculated as 1.31, 1.53, 1.74, 1.40, and 1.32 kg DM per kg shrunk BW.

**Table 2.** Teff yield (kg/ha) during the 63-d grazing periods as affected by beef steers supplement type and grazing day

	Grazing day													
	0		14		28		42		56			P-value^3^		
Item^1^	Control^2^	DDGS^2^	Control	DDGS	Control	DDGS	Control	DDGS	Control	DDGS	SEM	S	D	S × D
DM	0.706	0.849	0.954	0.971	1.157	1.157	0.811	1.163	0.993	0.945	0.269	0.43	0.38	0.83
OM	0.661	0.790	0.881	0.891	1.085	1.073	0.760	1.087	0.940	0.893	0.253	0.46	0.39	0.83
NDF^4^	0.472	0.563	0.661	0.677	0.783	0.797	0.483	0.662	0.673	0.650	0.181	0.48	0.01	0.94
ADF^5^	0.200	0.234	0.302	0.310	0.352	0.370	0.251	0.373	0.332	0.316	0.138	0.38	0.01	0.83
CP^6^	0.059	0.061	0.065	0.068	0.074	0.069	0.084	0.094	0.127	0.115	0.037	0.98	0.02	0.99
IVTDMD, %	0.450	0.540	0.567	0.579	0.700	0.661	0.532	0.713	0.676	0.644	0.177	0.58	0.55	0.89
IVTOMD, %	0.422	0.504	0.525	0.533	0.658	0.615	0.502	0.669	0.644	0.612	0.168	0.62	0.52	0.89

^1^DM: dry matter; OM: organic matter; NDF: neutral detergent fiber; ADF: acid detergent fiber; CP: crude protein; IVTDMD: in vitro true dry matter digestibility; IVTOMD: in vitro true organic matter digestibility.

^2^Control: cottonseed meal, 0.45 kg DM/animal-daily; DDGS: dried distillers grains plus solubles, 0.50% mean paddock BW/animal-daily.

^3^S: supplement type; D: grazing day; S × D: supplement type × grazing day interaction.

^4^Measure at d 42 lower (*P* ≤ 0.0001) than all other d; no other differences (*P* ≥ 0.70).

^5^Measure at d 0 lower (*P* ≤ 0.004) than all other d; d 56 higher (*P* ≤ 0.0002) than all other d; no other differences (*P* ≥ 0.33).

^6^Measure at d 28 lower (*P* = 0.04) than d 56 and tended (*P* = 0.14) to be lower than d 42; d 56 higher than d 0 and 14 (both, *P* = 0.02); no other differences (*P* ≤ 0.16).

Theoretically, estimating nutritive value of forage quality consumed in a grazed monoculture would be more reliable than in a multispecies culture (Rouquete, 2016). Along with level of intake, nutrient digestibility is a primary factor in establishing the upper threshold of potential animal weight gain (Sollenberger and Vanzant, 2011). In the current experiment, neither supplement type (*P* = 0.58) nor grazing day (*P* = 0.55) had a significant effect on estimated IVTDMD or IVTOMD of teff. Using six warm-season perennial grasses, Duble et al. (1971) described the inverse relationship of forage IVDMD to forage allowance required to realize maximum BW gain. When possible, analysis of canopy components representative of those selected by the animal are more appropriate than entire canopy when incorporating nutritive value as a function of forage mass (Roth et al., 1990). However, in the absence of a canopy component effect (*P* = 0.18, Table 2) on IVTDMD or IVTOMD, it would appear that digestibility measures of teff could simply be obtained from full tillers.

In vitro true DM digestibility of teff ranged from 63.3% to 66.3% over the grazing season. These measures are relatively consistent as indicated by the absence of a grazing day effect (*P* = 0.50) and equate to an approximate mean availability of 628 kg of digestible DM/ha across the grazing season. This sustained level indicates that teff could support ADG of growing beef cattle on the magnitude of 1.0 to 1.2 kg/d at forage mass allowances of approximately 750 kg/ha, or less, according the relationships described by Duble et al. (1971).

Beretta et al. (2006) assessed cattle grazing summer pastures of mixed legume and grass (*Lotus corniculatus* and *Festuca arundinacea*) and reported that beef steer intake would increase in response to increased forage allowance up to the greatest allowance observed of 9 kg per 100 kg BW. However, when supplemented with 0.45 kg of cracked corn per animal-daily, increases in forage allowance did not result in greater BW gain over the course of the grazing period relative to non-supplemented steers with a forage allowance of 9 kg DM per 100 kg BW. These findings may have resulted from a reduction of forage digestion associated with the corn supplement. Nevertheless, Beretta et al. (2006) maintained that maximization of BW gain per area may be achieved through a combination of increased stocking rate and supplementation. Regarding the consistency of the digestibility of teff observed, supplementation regimens might require fewer formulation adjustments during the course of the grazing period.

Considering that forage digestibility generally decreases with maturity, it could be hypothesized that the stability of teff digestibility across a grazing season would result in a consistent rate of forage DMI provided adequate forage mass. As a result, non-ammonia N flow expressed as g per g of dietary N may remain constant over the course of the grazing period (Losada et al., 1982). If so, this would contribute to the likelihood that supplementation could be simplified in that animal growth performance and teff forage utilization over the entire grazing season could be maintained without changes to rate or formulation of supplements.

### Steer Growth Performance

Steers provided DDGS gained 0.22 kg per d more (*P* = 0.01) than steers offered Control treatment over the 63-d grazing period (Table 3). The increased weight gain was not unexpected given the greater energy value and higher feeding rate of DDGS relative to cattle assigned to Control treatment. Higher energy values and associated increases in ADG by cattle fed dried distillers grains were likely due to increased energy from collective concentration of fat, metabolizable protein, and digestible fiber (MacDonald et al., 2007).

**Table 3.** Beef steers growth performance and dietary nutrient input predictions during the 63-d grazing periods as affected by supplement type

		Supplement type^1^			
Item^2^		Control	DDGS	SEM	P-value
Initial BW, kg		290	286	1.9	0.19
Final BW, kg		362	373	4.3	0.06
BW gain per ha, kg		147	176	7.0	0.01
ADG, kg					
	d 0–21	1.02	1.24	0.12	0.13
	d 21–42	1.47	1.71	0.13	0.11
	d 42–63	0.97	1.18	0.13	0.16
	d 0–63	1.16	1.38	0.06	0.01
Model predictions^3^					
	Teff TDN, %	58.38	54.98	0.93	0.01
	Teff NE_m_, Mcal/kg	1.31	1.20	0.03	0.02
	Teff NE_g_, Mcal/kg	0.73	0.63	0.03	0.03
	Teff DMI, kg	7.04	5.92	0.04	<0.01
	Total DMI, kg	7.60	7.68	0.05	0.14
	Diet NE_m_, Mcal/kg	1.31	1.40	0.02	0.01
	Diet NE_g_, Mcal/kg	0.74	0.82	0.02	0.02
	Gain:feed	0.151	0.182	0.009	0.01

^1^Control: cottonseed meal, 0.45 kg DM/animal-daily; DDGS: dried distillers grains plus solubles, 0.50% mean paddock BW/animal-daily.

^2^BW: body weight; ADG: average daily gain; TDN: total digestible nutrients; NE_m_: net energy for maintenance; NE_g_: net energy for gain.

^3^Beef Cattle Nutrient Requirements Model, 2016. National Academies Press. Washington, DC.

Predictions of teff energy concentration were derived using observed gain performance and predicted teff DMI. In estimating these energy values, default tabular measures of supplement TDN (69.65% and 89.00% for cottonseed meal and dried distillers grains, respectively) were used (NASEM, 2016). As a percentage of shrunk BW, simulated teff DMI was 2.16% and 1.80% for steers assigned Control and DDGS, respectively. However, total-diet predicted DMI as a percentage of shrunk BW was 2.33% for both treatments. Greater NE values for Control-teff relative to DDGS-teff are the product of greater TDN (*P* = 0.01) of the Control-teff combination. Supplementation, particularly with DDGS at a rate of 0.5% of shrunk BW, should result in forage substitution which would reduce forage intake (Morris et al., 2005, 2006; Griffin et al., 2012). Loy et al. (2007) fed DDGS at a rate of 0.4% BW to heifers consuming chopped smooth bromegrass grass hay and reported a daily forage DMI reduction of 0.21% BW. Similarly, MacDonald et al. (2007) found that heifers reduced DMI of smooth bromegrass pasture (*Bromus inermis*) by 0.45 to 0.50 kg per kg DDGS consumed depending on the method used to estimate pasture intake. In these and other studies, findings are consistent and indicate that DDGS supplemented at rates from 0.40% to 0.60% BW to growing cattle on forage-based diets may reduce forage DM consumption at a magnitude equal to one-half the rate of supplementation. In this study, model-predicted teff DMI by steers provided DDGS was 16% lower than by steers offered the Control treatment (*P* ≤ 0.01).

### Forage Nutrient Utilization and Water Footprint

Model-predicted teff DMI and mean nutrient concentrations across the grazing period were used to estimate total nutrient utilization (Table 4). With the exception of CP, calculated nutrient utilization aligned with greater predicted forage DMI by steers offered the Control treatment. When standing forage was sampled for the final time on d 56, no differences in nutrient yield were observed (*P* ≥ 0.33). When assessing the relationship between observed ADG and apparent nutrient utilization, it appears that there was an improvement in production efficiency, that is, in total weight gain per unit area forage mass, when steers were supplemented with DDGS. Simultaneously, one interpretation of the apparent lesser nutrient utilization by steers offered DDGS combined with similar measures of residual teff between the treatment groups could be that less teff was produced in paddocks supplemented with DDGS. However, given that agronomic procedures and climatic factors were constant across all paddocks, it seems more likely that similarity in nutritive yield at the termination of the trial is explained by an alternative reason. For instance, steers supplemented with DDGS could have exercised a greater degree of selective grazing due to a greater allowance resulting from lesser forage DMI. The subject of preferential selection has been previously described (Provenza et al., 2003). Such a response would accommodate the increased growth performance of steers provided DDGS due to the higher level of CP found in the grazed canopy of teff.

**Table 4.** Estimated teff nutrients consumed (through d 56) and residual teff nutrient mass available following a 63-d grazing periods by supplemented beef steers

		Supplement type^1^			
Item^2^		Control	DDGS	SEM	P-value
Nutrient utilization^3^					
	DM, t/ha	2.002	1.824	0.063	0.05
	OM, t/ha	1.920	1.745	0.087	0.05
	NDF, t/ha	1.350	1.239	0.031	0.04
	ADF, t/ha	0.625	0.571	0.016	0.04
	CP, t/ha	0.238	0.219	0.065	0.32
	Digestible DM, t/ha	1.403	1.302	0.107	0.12
	Digestible OM, t/ha	1.350	1.250	0.122	0.10
	NE_m_, Mcal/ha	2,622	2,188	79	<0.01
	NE_g_, Mcal/ha	1,461	1,149	43	<0.01
Residual nutrient yield^4^					
	DM, t/ha	0.993	0.945	0.164	0.35
	OM, t/ha	0.940	0.893	0.188	0.34
	NDF, t/ha	0.673	0.650	0.080	0.51
	ADF, t/ha	0.332	0.316	0.034	0.33
	CP, t/ha	0.137	0.131	0.075	0.73
	Digestible DM, t/ha	0.666	0.661	0.185	0.87
	Digestible OM, t/ha	0.634	0.626	0.197	0.79

^1^Control: cottonseed meal, 0.45 kg DM/animal-daily; DDGS: dried distillers grains plus solubles, 0.50% mean paddock BW/animal-daily.

^2^DM: dry matter; OM: organic matter; NDF: neutral detergent fiber; ADF: acid detergent fiber; CP: crude protein; NEm: net energy for maintenance; NEg: net energy for gain.

^3^Calculated from model predicted intake and mean nutrient concentration across sampling days.

^4^Calculated from forage mass availability and nutrient concentration.

Estimated water footprint values are presented in Table 5. Despite the increased ADG on paddocks assigned DDGS, stocking rates expressed as AUD per ha did not differ by supplement treatment (*P* = 0.67). Similarly, when water received was incorporated into the assessment, no difference in m^3^ per AUD per ha were observed (*P* = 0.32). Across years, paddocks received an average of 203.3 ± 40 m^3^ of irrigation per ha, and mean rainfall was 98.5 m^3^ per ha (Figure 1). When irrigation and rainfall per ha were summed and divided by mean total kg shrunk BW gain per ha, the resulting water footprint values equaled 1.76 and 1.96 m^3^/kg for Control and DDGS, respectively. These values were not subjected to statistical analysis. However, the estimates are slightly lower than the values of 2.9 to 4.8 produced from irrigation plus drinking water consumed by steers grazing grass pastures containing 21% teff (Baxter et al., 2017). The reduction in footprint values in the current study are likely due primarily to provided supplementation for which associated water contributions were not quantified. Other figures for water use relative to unit of beef production have been published (Beckett and Oltjen, 1993; Rotz et al., 2015). However, a lack of uniformity in considered inputs and scope of production make useful comparisons difficult.

**Table 5.** Water footprint (volume of water received per unit of each component output^1^) of teff grass during the 63-d grazing periods as affected by beef steers supplement type

	Supplement type^2^			
Item^3^	Control	DDGS	SEM	P-value
DM, m^3^/kg	0.09	0.12	0.03	0.14
OM, m^3^/kg	0.09	0.12	0.02	0.19
NDF, m^3^/kg	0.13	0.18	0.03	0.17
ADF, m^3^/kg	0.29	0.39	0.07	0.19
CP, m^3^/kg	0.68	0.89	0.12	0.16
Digestible DM, m^3^/kg	0.12	0.17	0.03	0.18
Digestible OM, m^3^/kg	0.13	0.17	0.03	0.23
NE_m_, m^3^/Mcal	0.07	0.10	0.02	0.15
NE_g_, m^3^/Mcal	0.12	0.19	0.04	0.12
AUD/ha	93.39	94.21	5.51	0.67
m^3^/AUD-ha	1.85	2.28	1.26	0.32

^1^Calculated as nutrient composition multiplied by the predicted steer DMI during the entire grazing period plus nutrient concentration at final sampling (d 56); Estimates of NE_m_ and NE_g_ were derived from estimated steer DMI.

^2^Control: cottonseed meal, 0.45 kg DM/animal-daily; DDGS: dried distillers grains plus solubles, 0.50% mean paddock BW/animal-daily.

^3^DM: dry matter; OM: organic matter; NDF: neutral detergent fiber; ADF: acid detergent fiber; CP: crude protein; NE_m_: net energy for maintenance; NE_g_: net energy for maintenance; AUD: animal unit days.

**Figure 2.**
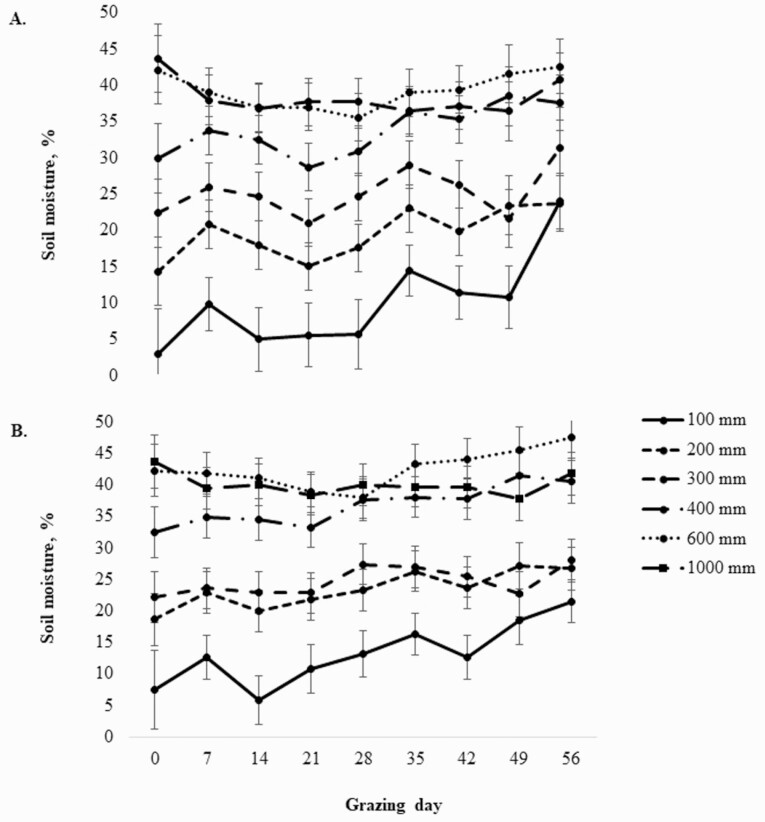
Teff paddocks soil moisture (%) during the 63-d grazing periods as affected by beef steers supplement treatment (Control-cottonseed meal [**A**] or dried distillers grains plus solubles [**B**]). Soil moisture did not reflect a supplement × soil depth × grazing day interaction (*P* > 0.99); neither supplement × grazing day (*P* = 0.89) nor supplement × soil depth (*P* = 0.20) interactions were detected. Differences in soil moisture by soil depth (*P* < 0.01) and grazing day (*P* < 0.01) are depicted in Table 5.

In regard to soil water content, no interactions of supplement type × day × soil depth (*P* > 0.99), supplement type × day (*P* = 0.89), supplement type × soil depth (*P* = 0.20), or soil depth × day (*P* = 0.24) on soil water content was observed (Table 6). Soil water content by supplement and day are depicted in Figure 2. No specified requirements for soil water content related to teff production can be found in the literature. In addition to differences among cultivar, root depths of teff grain crops have been reported to range from 59 to 100 cm in drought scenarios and 86 to 116 cm when aided by irrigation (Ayele et al., 2001). However, root biomass of teff is predominately concentrated in the upper 30 cm of the soil surface with actual depths correlated with plant height (Ayele et al., 2001). Given that stocking was continuous in this trial, plant height was kept short. Therefore, it can be stated that moisture level in this experiment at a depth of 30 mm (25%) was sufficient to maintain a stand of teff throughout the grazing season.

**Table 6.** Soil moisture (%) during the 63-d grazing periods as affected by beef steers supplement treatment, soil depth, and grazing days

Supplement type^1^		
	Control	27.79
	DDGS	30.10
	SEM	2.71
Soil depth, mm		
	100	12.38^a^
	200	21.61^b^
	300	25.26^c^
	400	35.34^d^
	600	40.69^e^
	1000	38.38^e^
	SEM	1.01
Grazing day		
	0	27.11^wxy^
	7	28.52^wxy^
	14	26.81^wx^
	21	26.07^w^
	28	27.71^wxy^
	35	30.70^yz^
	42	29.45^xy^
	49	30.51^xyz^
	56	33.63^z^
	SEM	1.22
*P*-values		
	Supplement type	0.40
	Soil depth	<0.01
	Grazing day	<0.01
	Supplement type × soil depth	0.27
	Supplement type × grazing day	0.87
	Soil depth × grazing day	0.39
	Supplement type × soil depth × grazing day	>0.99

^1^Control: cottonseed meal, 0.45 kg DM/animal-daily; DDGS: dried distillers grains plus solubles, 0.50% mean paddock BW/animal-daily.

^a–e^Means without a common superscript differ *P* ≤ 0.05.

^w–z^Means without a common superscript differ *P* ≤ 0.05.

## CONCLUSIONS

Nutrient yield and water use efficiency of continuously stocked teff grass was not affected by supplemental regimen. Using DDGS as a supplement may increase BW gain through increased nutrient utilization without hindering teff nutrient production on a teff continuous stocking system. Teff maintained a consistent level of digestibility over the growing season and only generated differences in detergent fiber concentrations between portions of the canopy structure near the end of the growing season. It appears that choice of supplement to continuously stocked teff would neither be expected to influence forage nutrient yield following the grazing period nor amount of water needed to produce BW gain throughout the period. Teff grass should be considered as a practical annual-forage option for early summer to early fall stocker production with total system output determined by choice of supplement regimen employed to achieve desired body weight gain by grazing cattle.

## CONFLICT OF INTEREST

The authors report no conflicts of interest.
